# The Musculoskeletal Pain Intensity and Interference Questionnaire for Musicians: Assessment of Patient-Related Outcomes among Professional Orchestra Musicians in Poland—A Cross-Sectional Study

**DOI:** 10.3390/jcm13164751

**Published:** 2024-08-13

**Authors:** Anna Katarzyna Cygańska, Michał Kaczorowski, Beata Rodzik

**Affiliations:** 1 Faculty of Rehabilitation, Jozef Pilsudski University of Physical Education in Warsaw, Marymoncka 34, 00-968 Warsaw, Poland; michal.kaczorowski@awf.edu.pl; 2 Faculty of Mathematics, Maria Curie-Skłodowska University, Plac Marii Curie-Skłodowskiej 1, 20-031 Lublin, Poland; beata.rodzik@gmail.com

**Keywords:** playing-related musculoskeletal disorders, musicians, musculoskeletal pain, questionnaire, pain

## Abstract

**Background:** Musculoskeletal pain is one of the reasons for a musician’s inability to play an instrument. An assessment of the localization, intensity, and interference of those complaints is important among musicians because of the risk of occupational disease. Assessment by validated tools is especially important and serves as an indicator to take up proper preventive or treatment actions. The aim of the study was to assess the prevalence of playing-related musculoskeletal disorders (PRMDs) and the relationships with PRMDs’ impact on playing musical instruments among Polish professional orchestra musicians. **Methods:** The study was conducted on a group of professional orchestra musicians (age 37.19 ± 9.62 years), 99 (55%) women and 83 (45%) men. Work experience in professional orchestra was 18.3 ± 9.83 years and the reported years of playing musical instrument were 31.4 ± 9.50. The study used the online version of a musician-dedicated questionnaire, Musculoskeletal Pain Intensity and Interference Questionnaire for Musicians of the Polish Population (MPIIQM-P). **Results:** In the study group, 89.9% of women and 83.1% of men had experienced playing-related pain in their lifetime. The most intense pain among the group with current playing-related musculoskeletal complaints (*n* = 85) was located in the neck area for 19 (22.4%). **Conclusions:** The results of the study indicate a high prevalence of playing-related musculoskeletal problems among professional musicians.

## 1. Introduction

Playing-related musculoskeletal disorders (PRMDs) are defined as “any pain, weakness, numbness, tingling or other physical symptoms that interfere with your ability to play your instrument at the level you are accustomed” [1]. Musculoskeletal disorders affect professional musicians more often than other professional groups [2,3], and among professional musicians, women experience pain related to playing an instrument more often than men do [4]. During their professional career, up to 90% of musicians experience playing-related musculoskeletal pain [5], and between 49% and 86.5% of the musicians experienced an episode of pain during the month preceding the study [6,7,8]. Pain contributes to discomfort during playing for up to 54% of orchestral musicians, with 18% of them taking sick leave due to their inability to play [9]. The occurrence of pain in this professional group results from the combination of multiple physical and psychological factors. The musicians’ physical strain stems from having to maintain a demanding and asymmetrical position for many hours, countlessly performing quick, precise and repetitive movements [10]. The psychological stress that contributes to the occurrence of the episodes of pain is caused, among others, by stage fright [5,10,11]. As a consequence, up to 43.4% of musicians suffer from pain in more than five areas of the body [10]. The musculoskeletal pain experienced by the professional musicians is most often located in the shoulder girdle and neck [12]. Furthermore, the study highlights the body regions most frequently afflicted with pain among musicians playing various instrument groups, given that each instrument demands distinct body postures and specific movements during performance [5].

To the best of our knowledge, no studies have been conducted in Poland to assess patient-related outcomes in the population of Polish professional orchestral musicians. Currently, there are no data on playing-related musculoskeletal disorders among professional orchestral musicians in Poland.

The aim of the study was to assess patient-related outcomes as the prevalence of playing-related musculoskeletal disorders (PRMDs), collecting information on the location and intensity of pain and the relationships with PRMDs’ impact on playing musical instruments among professional orchestra musicians in Poland.

## 2. Materials and Methods

All musicians playing at 10 Polish symphony orchestras (*n* = 848) were invited to participate. The number of professional musicians working in individual orchestras was confirmed by each orchestra’s administration.

In total, 182 (aged 37.19 ± 9.62 years) of 848 musicians completed the questionnaire, including 99 (55%) women and 83 (45%) men. Out of 182 respondents, 85 answered “yes” to questions 11 and/or 12 (pain in the past 7 days), therefore being able to complete the subsequent part of the MPIIQM-P questionnaire. All respondents gave informed consent to partake in the study, having been informed that the participation was voluntary and anonymous.

The inclusion criteria for the study were: age over 18, Polish as the native language, and playing in a professional orchestra. Amateur musicians, musicians under the age of 18, and those with a native language other than Polish were excluded from the survey.

The study received a favorable opinion from the Senate’s Research Bioethics Committee (SKE 01-01/2023) and was conducted in accordance with the guidelines of the Declaration of Helsinki [13]. The study was registered at www.ClinicalTrials.gov, identification number: NCT05903300.

The MPIQIM questionnaire was used in the study. It was developed as a tool dedicated to professional musicians, taking into account the specificity of the studied group. The questionnaire has been translated into many languages both in paper and online versions. In Polish, it is available in both paper [14] and online [15] versions. The study used the online version of the MPIIQM-P questionnaire. The MPIIQM-P questionnaire consists of 22 items and provides the following data: demographic data (items 1 and 2), job-related data (items 3 through 8), frequency of pain over time (items 9 through 12), pain location (items 13 and 14), pain intensity (items 15 through 18), and pain interference (items 19 through 23). Pain intensity is determined using a numerical scale on which the extreme values are 0 (“no pain”) and 10 (“the most severe pain”). Pain interference was also measured on an 11-degree numerical scale with the same numerical range, and the extremes in items 19 and 20 are described as 0 (“did not interfere”) and 10 (“completely interfered”), and in items 21 through 23, 0 (“no difficulty”) and 10 (“unable”). The overall level that determines pain intensity (max. 40 points) and pain interference (max. 50 points) is calculated based on the sum of the values obtained for each item, and the total score of the questionnaire is 90 points [14]. Access to the questionnaires was sent to the musicians via email by the administrations of the individual orchestras. The data collection lasted from January 2024 to May 2024.

### Statistical Analysis

Based on a sample size calculation using G*Power (17 March 2020—Release 3.1.9.7), it is estimated that male and female musicians have a pain prevalence of 60% and 85%, respectively [16]. An effect size of 0.6 was used, with an alpha error probability of 0.05 and a power (1 − β) of 0.80. Therefore, the minimum sample size required was 74 musicians. Quantitative data were presented using the following measures: arithmetic mean and standard deviation. Qualitative data were presented in a percentage format. The normal distribution was calculated with the use of the Shapiro–Wilk test. Data were not normally distributed, so the U Mann–Whitney test and Chi square test were used to evaluate differences between different sex groups, and groups with or without the presence of musculoskeletal pain symptomatology. The statistical significance level was set at *p* < 0.05. Statistical analyses were performed using STATISTICA 13 (TIBCO Software Inc., Palo Alto, CA, USA).

## 3. Results

The professional experience of the musicians amounted to 11.77 ± 9.66 years, and their experience of playing their main instrument equaled to 27 ± 10 years. In total, 182 musicians submitted their responses (21% response rate). Out of 182 respondents, 85 answered “yes” to questions 11 and/or 12, thus being able to complete the subsequent part of the MPIIQM-P questionnaire.

Musicians reported experiencing playing-related musculoskeletal problems as follows: 158 (86.8%) in their lifetime, 83 (45.6%) within the past 12 months, 71 (39%) within the past 4 weeks, and 59 (32.4%) currently (within the past 7 days). Detailed characteristics of the study group divided by gender are presented in Table 1.

The study group consisted of individuals, 66.5% (*n* = 121) of whom were string players, 14.8% (*n* = 27) were brass players, 13.7% (*n* = 25) were woodwind players, and 4.4% (*n* = 8) were percussion players; one person played the piano (0.5%). A detailed breakdown of the instruments played by the study participants is presented in Table 2.

The most intense pain among the group with current playing-related musculoskeletal complaints (*n* = 85) was located as follows: in the neck area for 19 (22.4%) musicians, in the thoracic spine for 16 (18.8%), in the right shoulder for 14 (16.5%), in the lumbar spine for 9 (10.6%), in the right forearm for 7 (8.2%), in the right hand for 6 (7%), in the left shoulder for 3 (3.5%), in the left hand for 3 (3.5%), in the head area for 2 (2.4%), in the right wrist for 2 (2.4%), in the right arm for 1 (1.2%), in the left forearm for 1 (1.2%), in the left wrist for 1 (1.2%), and in the right thigh for 1 (1.2%).

From what was observed, there is a significant relationship between age (*p* = 0.032) and the amount of time spent playing the instrument per week (*p* = 0.005) in regard to the occurrence of current pain. Participants experiencing current pain were older (38.83 ± 10.35 vs. 35.75 ± 8.73) and were spending more hours playing an instrument in an orchestra (22.34 ± 7.74 vs. 19.73 ± 7.08) compared to musicians not suffering from current pain. Detailed characteristics of the study group divided into musicians who reported current pain and those who did not have any pain are presented in Table 3.

In order to determine the locations of pain characteristic of the players of a given type of instrument, the musicians were divided into groups. Five groups of instruments were distinguished: upper string (violin and viola), lower string (double bass and cello), brass (horn, trumpet, trombone, and tuba), woodwind (flute, oboe, clarinet, and bassoon) and percussion instruments. The “percussion instruments” group was excluded from the statistical analysis due to the insufficient number of respondents (*n* = 2). A difference in the location of PRMD, observed in the study, depends on the instruments played by professional orchestral musicians. Among the upper string players, the most frequently declared location of PRMD was the cervical spine, as well as both the back and the front of the right shoulder. In the group of lower string players, the three dominant locations of PRMD were the upper back, the lower back, and the neck. For the group of brass players, the locations of pain were the upper back, the front of the right shoulder, and the face, while for the woodwind players, the indicated areas were the cervical spine and the front of the right shoulder. The third most common location of PRMD among woodwind players was not indicated, as there was no uniformity (multiple separate areas were reported by the individual respondents) in the respondents’ answers; therefore, the data were insufficient. The detailed results of the analysis are presented in Table 4.

The lifetime prevalence of playing-related musculoskeletal problems was determined for five separate groups of instrumental musicians. In the group of upper string players, the prevalence of PRMD was 91.11%, reaching 93.55% in the group of lower string players, whereas for the brass players, PRMD amounted to 77.78%, and to 80% for the woodwind players. The results are presented in Table 5. Due to the insufficient number of respondents (*n* = 2), the “percussion instrument” group is not presented in Table 5.

## 4. Discussion

In the conducted study, 89.9% of women and 83.1% of men had experienced playing-related pain in their lifetime. The obtained results were comparable to the results which were already presented in the literature on this subject, according to which the occurrence of lifetime prevalence ranges from 46% to 90% among the respondents [5].

This study revealed that the musicians experiencing current musculoskeletal pain were older and played the instrument for a longer amount of time per week. It might also be related to a natural career development and a greater workload for more experienced musicians. Therefore, longer playing time due to the musicians’ professional duties results in a higher load of muscles. In addition, the body’s ability to regenerate decreases with age. However, the scientific literature was not concordant with the described correlation. There are studies that have shown a correlation between the musicians’ age, the time devoted to playing the instrument per week, and pain [17,18]; however there are also studies that have not presented such correlation [19,20].

The most common location of musculoskeletal pain (the neck for 22.4% of respondents with PRMD) among professional musicians shown in the study confirms the results obtained by other researchers. Gómez-Rodríguez et al. [21] showed that during the 7 days preceding the study, 45.5% of the respondents experienced neck pain, and 31.7% of them experienced shoulder pain. Kochem et al. [8] showed that in the previous 12 months, the most common locations of pain for the responders were the neck (56.6%), the upper back (49.1%), and both the right (45.3%) and left (44.3%) shoulders. Steinmetz et al. [10] showed that the pain in the neck area occurred most commonly, regardless of the instrument played by the musicians. The pain of this area affected from 53.8% to 76.7% of professional musicians.

The locations of musculoskeletal pain shown in this study for the groups of musicians playing upper string, lower string, brass, and woodwind instruments align with the existing literature. Van Selms et al. [18] also showed that the neck and shoulders were the most common locations of PRMD among upper string musicians. Vastamäki et al. [3] showed that among professional orchestral musicians, the back was the most common location of PRMD. Van Slems et al. [18] reported that 15.4% of brass players experienced craniofacial pain, which complies with the data obtained in this study, equal to 13.3% for this particular group of musicians. Raising the upper limbs while playing string instruments affects the load of both shoulder girdle and shoulder muscles. Perhaps as a consequence of the musicians having to assume different positions when playing an instrument, they report the occurrence of PRMD in separate locations, related to the particular postures. A biomechanical study conducted by Hildebrandt et al. [22] showed the impact of the position taken while playing upper string instruments on muscle work and fatigue felt by the musicians. It was shown that reducing the inclination angle of the instrument in relation to the frontal plane from 50 degrees to 20 degrees, as well as reducing the lateral orientation angle (determined between the vertical axis of the instrument and the musician’s sagittal plane) from 50 degrees to 20 degrees, increases the activation of shoulder girdle muscles, measured by EMG and the respondents’ subjective feeling of fatigue. Cruder et al. [23] showed that the musicians who have to raise both upper limbs while playing an instrument (e.g., trumpeters and trombonists) declared the highest incidence of PRMD, equal to 54.4%. Moreover, for the musicians playing with one upper limb raised (e.g., double bassists and cellists) the incidence was 51.1%, while the instrumentalists not having to raise upper limbs when playing (e.g., drummers) declared an incidence of PRMD of 47.7%. This study showed that the most common location of PRMD for the group of brass players was the upper back. Pain in the upper back might be a consequence of maintaining a standing position while playing an instrument. Rodriguez-Romero et al. [20] showed that musicians who maintain a standing position while playing an instrument were more likely to experience pain in the upper back and lower back. According to the results obtained in this study, the third most common location of PRMD among brass players was the craniofacial region. Playing wind instruments requires a significant effort of the facial muscles, which can lead to strain and pain within these muscles and temporomandibular joints [24,25].

It was shown that brass players had the lowest lifetime prevalence of pain, equal to 77.78%, which is in line with the existing literature. Steinmetz et al. [10] showed that the prevalence of PRMD in a lifetime among professional musicians equaled to 90.3% in the group of upper string players, 90.6% in the group of lower string players, 86.9% in the group of woodwind players, and 83.6% in the group of brass players. Kok et al. [26] showed that the prevalence of PRMD in the 12 months preceding their study amounted to 93% for woodwind players and 86% for brass players. The analysis showed that musicians playing lower string instruments (93.55%) and upper string instruments (91.11%) were the ones who most commonly experienced playing-related pain during their lifetime. Unfortunately, based on the existing literature, it is not possible to clearly determine which of the groups of instrumentalists is at a highest risk of PRMD, due to the significant heterogeneity of the research [27].

In the conducted study, women were more likely to experience playing-related pain throughout lifetime. When playing an instrument, the burdens associated with the piece being played are the same for each gender. It is possible that the more frequent musculoskeletal complaints in women are due to biological, gender-specific factors. One of these are sex hormones that can affect the perception of pain. Estrogen fluctuations are one of the factors that lower the pain threshold and thus predispose to more frequent complaints [28]. Another factor may be differences in muscle characteristics and strength. Deodato et al. [29] showed that in the same study group, women scored worse than men on tests of muscular endurance. The gender-independent stresses associated with playing an instrument combined with lower muscular endurance among women may predispose them to more frequent strains and consequent pain.

The conducted study exhibits strengths. Thanks to the use of the MPIIQM-P questionnaire in its electronic form, a large study group (*n* = 182) was involved. Using the electronic form of the questionnaire allowed us to reach a much larger study group than in the case of distributing the questionnaires in paper form. However, research in electronic form is characterized by a lower response rate. In our study, the response rate was 21%, and therefore the findings may not be generalizable to all the musicians.

The main limitations of the conducted survey are voluntary bias and the fact that the data were based on a self-reported questionnaire. In subsequent studies, the results based on a self-reported questionnaire could be compared to clinical evaluation by a medical specialist.

The necessity to identify factors that affect pain results from the multifactorial etiology of PRMD. Identifying further variables affecting PRMD aside from time, the intensity of playing, or the type of instrument, will enable the introduction of effective preventive measures. Subsequent studies should determine the physical activity level among professional musicians, as well as providing information about the level of their health education, the physiotherapy undergone by them, and other preventive measures taken that could affect the prevalence of PRMD. In addition, future research should examine the muscular endurance of the muscle groups involved in playing the instrument. The data obtained may make it possible to determine whether muscular endurance is an important factor in determining the onset of musculoskeletal complaints.

## 5. Conclusions

1. Playing-related musculoskeletal disorders are frequent among professional orchestral musicians of both sexes.
2. Musicians experiencing PRMD were older and play the instrument for a longer period of time during a week due to their duties in the orchestra.
3. The most common locations of playing-related musculoskeletal disorders were the neck, the shoulder girdle, and the upper back.

## Figures and Tables

**Table 1:** Characteristics of the study group and prevalence of PRMD by gender (M ± SD).

Item	Women N = 99	Men N = 83	*p*-Value
Age (years)	36.69 ± 8.73	37.80 ± 10.62	0.598 ^1^
Playing time on the instrument (years)	27.41 ± 8.95	26.65 ± 11.19	0.365 ^1^
Playing time in the orchestra (years)	11.22 ± 8.81	12.44 ± 10.62	0.797 ^1^
Weekly hours of playing in the orchestra	21.13 ± 6.69	20.73 ± 8.39	0.617 ^1^
Weekly hours of playing outside the orchestra	12.25 ± 7.48	12.80 ± 8.27	0.768 ^1^
Prevalence of playing-related musculoskeletal pain (*n*, %)			
Lifetime prevalence	89 (89.9%)	69 (83.1%)	0.179 ^2^
Past 12 months	46 (46.5%)	36 (43.4%)	0.676 ^2^
Past 4 weeks	33 (33.3%)	37 (44.6%)	0.120 ^2^
Past 7 days	25 (25.6%)	33 (39.8%)	0.037 ^2^*

* *p* < 0.005; ^1^—Mann–Whitney U test; ^2^—Chi square test of independence.

**Table 2:** Characteristics of the study group by instrument (*n*, %).

Instrument Type	Number of Participants (*n*, %)
String	121 (66.5%)
Violin	68 (37.4%)
Viola	22 (12.1%)
Cello	16 (8.8%)
Double bass	15 (8.2%)
Woodwind	27 (14.8%)
Flute	9 (4.9%)
Oboe	5 (2.7%)
Clarinet	4 (2.2%)
Bassoon	7 (3.8%)
Brass	25 (13.7%)
French horn	7 (3.8%)
Trumpet	12 (6.7%)
Trombone	6 (3.3%)
Tube	2 (1.1%)
Percussion	8 (4.4%)
Percussion	8 (4.4%)
Other	1 (0.5%)
Piano	1 (0.5%)

**Table 3:** Characteristics of the study group divided into musicians with PRMD (in the past 7 days) and without PRMD (M ± SD).

Item	Musicians with PRMD *n* = 85	Musicians without PRMD *n* = 97	*p*-Value
Age (years)	38.83 ± 10.35	35.75 ± 8.73	0.032 ^1^*
Playing time on the instrument (years)	28.09 ± 11	26.16 ± 8.97	0.220 ^1^
Playing time in the orchestra (years)	13.11 ± 10.28	10.6 ± 9.01	0.098 ^1^
Weekly hours of playing in the orchestra	22.34 ± 7.74	19.73 ± 7.08	0.005 ^1^*
Weekly hours of playing outside the orchestra	12.1 ± 8.09	12.78 ± 7.63	0.389 ^1^
Prevalence of playing-related musculoskeletal pain (*n*, %)			
Gender (F:M)	37 (43.5%):48 (56.5%)	62 (63.9%):35 (36.1%)	0.006 ^2^
Lifetime prevalence	85 (100%)	73 (75.3%)	<0.001 ^2^*
Past 12 months	53 (62.4%)	29 (29.9%)	<0.001 ^2^*
Past 4 weeks	70 (82.4%)	0 (0%)	<0.001 ^2^*
Past 7 days	58 (68.2%)	0 (0%)	<0.001 ^2^*

* *p* < 0.005; ^1^—Mann–Whitney U test; ^2^—Chi-square test of independence.

**Table 4:** PRMD location (past 30 days) by instrument group.

Instrument Type	Most Common Location of PRMD (*n*, %)	Second Most Common Location of PRMD (*n*, %)	Third Most Common Location of PRMD (*n*, %)
Upper string (*n* = 40)	Neck (10, 25%)	Right shoulder—back (6, 15%)	Right shoulder—front (6, 15%)
Lower string (*n* = 18)	Thoracic spine (7, 38.9%)	Lumbar spine (7, 38.9%)	Neck (3, 16.7%)
Brass (*n* = 15)	Thoracic spine (4, 26.7%)	Right shoulder—front (3, 20%)	Face (2, 13.3%)
Woodwind (*n* = 10)	Neck (4, 40%)	Right shoulder—front (3, 30%)	-

**Table 5:** Lifetime prevalence of PRMD by instrument group.

Instrument Group	Lifetime Occurrence of PRMD (*n*, %)	No Lifetime Occurrence of PRMD (*n*, %)
Upper string (*n* = 90)	82 (91.11%) ^1^	8 (8.89%) ^1^
Lower string (*n* = 31)	29 (93.55%) ^1^	2 (6.45%) ^1^
Brass (*n* = 27)	21 (77.78%) ^1^	6 (22.22%) ^1^
Woodwind (*n* = 25)	20 (80%) ^1^	5 (20%) ^1^

^1^—Pearson Chi-square test of independence; *p* < 0.05.

## Data Availability

Available upon request. Proposals should be directed to michal.kaczorowski@awf.edu.pl.
